# Evaluation of Biological, Textural, and Physicochemical Parameters of Panela Cheese Added with Probiotics

**DOI:** 10.3390/foods9101507

**Published:** 2020-10-21

**Authors:** Karina A. Parra-Ocampo, Sandra T. Martín-del-Campo, José G. Montejano-Gaitán, Rubén Zárraga-Alcántar, Anaberta Cardador-Martínez

**Affiliations:** Tecnologico de Monterrey, School of Engineering and Sciences, Epigmenio González 500, Fracc, San Pablo 76130, Querétaro, Mexico; karina.parraocampo@hotmail.com (K.A.P.-O.); smartinde@tec.mx (S.T.M.-d.-C.); gmonteja@tec.mx (J.G.M.-G.); rzarragaa@tec.mx (R.Z.-A.)

**Keywords:** panela cheese, angiotensin-converting enzyme inhibition, probiotic addition, antioxidant activity, DPPH, ABTS

## Abstract

Biological, physicochemical and textural parameters of a Panela cheese with and without probiotics (LSB-c and C-c) were analyzed during 15 days of storage at 4 °C. Changes in cohesiveness, hardness, springiness, and chewiness were measured by texture profile analysis. Additionally, moisture, pH, nitrogenous fractions (nitrogen soluble in pH 4.6, non-protein nitrogen, 70% ethanol-soluble nitrogen, and water-soluble extract) were evaluated. The peptide profile of nitrogenous fractions was also analyzed. Finally, biological activity was evaluated by ABTS (2,2′-Azino-bis(3-ethylbenzothiazoline-6-sulfonic acid) diammonium salt) and DPPH (2,2-diphenyl-1-picrylhydrazyl), as well as the Inhibition of Angiotensin-Converting Enzyme. Analysis of variance showed significant differences for most of the evaluated parameters. By principal component analysis (PCA), two groups were separated, one corresponding to LSB-c and the other corresponding to C-c. The separation was given mostly by hardness, chewiness, and ABTS of all nitrogenous fractions. LSB-c showed higher biological activities than C-c.

## 1. Introduction

Bioactive peptides are genuine or generated components of ready-to-eat foods that may exert a regulatory activity in the human organism, regardless of their nutritive functions [1].

It is known that bovine milk is the most significant source of food-derived bioactive peptides [2]. The existence of bioactive peptides in fermented milk products and ripened cheese has been described [3]. During proteolysis, various peptides are released from the milk proteins; they are inactive while encrypted in the milk proteins. Proteolysis takes place during food processing, e.g., milk fermentation and cheese maturation, or during gastrointestinal transit. Some of the bioactive properties reported in peptides derived from milk products are antihypertensive, antioxidant, antimicrobial, immunomodulatory, and mineral binding [4,5,6]. The amount and type of bioactive peptides in cheese are affected by the starter culture and ripening conditions [7].

In 2019, the production of fluid milk in Mexico was approximately 12.6 million metric tons, of which almost 50% was utilized for cheese elaboration [7]. According to SAGARPA (Mexican Ministry of Agriculture and Sustainable Development) [8], in 2018, Mexican cheese production was 418,650 tons, where panela represented the third most-produced cheese and represented 11.7% of total production.

Panela cheese is a very popular handcrafted Mexican white, soft, fresh cheese manufactured from pasteurized cow’s skim or partially skimmed milk [9], with little or no starter culture acidification [9,10]. According to the manufacture characteristics of this type of cheese, it is expected some degree of proteolysis and thus a release of bioactive peptides during manufacturing procedures, storage, and post-consumption.

On the other hand, hypertension is a state of a sustained increase in blood pressure (BP), related to cardiovascular diseases. Hypertension is the mortality most related factor around the world [11]. This is a chronic disease derived from many factors such as genetics, excessive sodium intake, age, smoking, sedentary lifestyle, and chronic diseases such as diabetes and obesity [12,13]. According to W.H.O. [14], more than one of every five adults suffers from hypertension.

Within the organism, the regulation of blood pressure is related to the hormone “renin-angiotensin system” (RAS). The angiotensin-converting enzyme (ACE) is key within RAS because it converts the peptide angiotensin I to the vasoconstrictor angiotensin II, which tightens the blood vessel and increase the BP. ACE-inhibitors are competitive substrates for ACE, and among them are milk-derived bioactive peptides. The C-terminal of the inhibitor is the primary feature governing the inhibition of ACE [11].

Oxidative stress is a condition of imbalance between reactive oxygen species (ROS) with unpaired electrons and the body’s ability to detoxify and repair the damage of the reactive components. It is widely related to the illnesses of the human body, including hypertension and other chronic diseases. Milk protein-derived peptides are among the natural dietary sources of antioxidants. Peptides from β-casein and α_s1_-casein are potent anion radical scavengers [11].

This work aimed to measure antioxidant, and ACE inhibition of a Panela cheese added (LSB-c)/not added (C-c) with probiotics. Physicochemical and textural parameters were also monitored during 15 days of storage (4 ± 0.6 °C) to evaluate their effect and relationship upon structural changes in both types of cheeses.

## 2. Materials and Methods

### 2.1. Cheese Manufacture

For this study, one 80-L batch of whole milk was obtained in the Tecnológico de Monterrey experimental agricultural field (CAETEC) (Querétaro, Mexico) and transported to the Tecnológico de Monterrey, Querétaro, Mexico under controlled temperature conditions. In the CAETEC, milk production is controlled to avoid composition variation throughout the year. To achieve this, the calf diet is standardized with a feed formulate by Tecnologico de Monterrey. The herd has about 100 milking cows. Different national associations and industrial clients have certified the homogeneity and quality of CAETEC milk.

Whole milk (3.11% protein, 3.19% fat, initial pH 6.69) was cooled and stored at 4 °C for 24 h before cheese making. For cheese making, the Querataro’s traditional panela making procedure was followed. Milk was pasteurized in a big pot (63 °C for 30 min) previously to cheese manufacturing and was split into two portions of 40-L at the food engineering facilities of Tecnológico de Monterrey, Querétaro, Mexico. The experimental design was a unifactorial design where the factor evaluated was storage time with four levels (0, 5, 10, and 15 days). Cheeses with probiotics (LSB-c) and without probiotics (C-c) where considered as independent blocks.

For the cheese added with probiotics (LSB-c), after pasteurization, the 40 L of milk was heated gradually to 32 °C, and 10^9^ CFU/L of commercial type MM101 (Lyofast^®^, Sacco, Via Manzoni, Italy) culture was added, which consisted of *Lactococcus lactis*, *Lactococcus cremoris*, *Lactococcus diacetylactis*, and *Streptococcus thermophilus*. Then, 10^9^ CFU/mL *Bifidobacterium animalis* ssp lactis (Lyofast^®^, Sacco, Via Manzoni, Italy) was added to the mix. Inoculated milk was kept at 32 °C for three h until it reached a pH value of 6.4. CaCl_2_ (Cal-Sol501, Industrias Cuamex, San Miguel Iztapalapa, CDMX, Mexico) was added by diluting 7 mL in 35 L of milk and kept in slow agitation for 1 min. Liquid calf rennet (Strength 1:7500. Qualact^®^, Altecsa SA, Mexico City, Mexico) was added to milk (1.95 mL + 30 mL water). Coagulation of milk was completed in 30–35 min; then, the curd was cut into cubes (1 cm^3^) and allowed to rest in whey for 5 min before draining approximately 2/3 of whey. Salt was added to curd at 1% and mixed manually for 5 min. Then cheeses were molded in 100 g plastic molds. Molds were slightly pressed for 20 min (by each side) by piling them up on one another, allowing natural whey drainage at room temperature. For C-c (control cheese, without any added culture), 40 L of milk were used following the above procedure without culture addition. Both kinds of cheeses were made simultaneously to avoid changes in milk quality. For each kind of cheese, 34 × 100 g-pieces were obtained, giving a total of 68 × 100 g pieces of cheese.

Individual cheeses were packed in plastic bags. Then, they were stored in refrigeration at 93.8 ± 1% of relative humidity (RH) and 4 ± 0.6 °C temperature for 15 days.

### 2.2. Sampling

Two whole 100-g pieces of cheese (LSB-c and C-c, respectively) were removed after 24 h (denoted as day 0), 5, 10, and 15 days of storage for biological, physicochemical, and textural determinations. Analysis was performed by triplicate on the same cheese, and two cheesemaking trials were done.

### 2.3. Physicochemical Analysis

Moisture was determined by oven drying cheese samples at 100 ± 2 °C, according to NOM-116-SSA1-1994 [15], by triplicate. The pH values of cheeses were determined on the surface by triplicate, according to NMX-F-317-S-1978 [16], with an Oakton pH meter (Eutech Instruments, Vernon Hills, IL, USA).

### 2.4. Instrumental Texture Profile Analysis

For texture properties evaluation, six replicates were made in Panela cheese samples with a CT3 Texture Analyzer^®^ (Brookfield, AMETEK, Middleborough, MA, USA). The texture was evaluated using a two-bite compression test. Cylindrical samples (1.8 cm of diameter and 1.5 cm height) were tested by using a 50 N load cell and two parallel plates (10 cm diameter). The compression ratio was established at 50% deformation from the original height and a rate of 200 mm/min, similar to a deformation rate between fingers during squeezing [17]. Cheeses were left at room temperature for 15 min after being removed from refrigeration before obtaining cheese cylinders and proceeding with the texture profile analysis (TPA). Parameters measured were cohesiveness, hardness, springiness, and chewiness, and were obtained from the force-time plots of Tension Zero version 1.0 [18]. Cohesiveness, defined as the strength of the internal bonds making up the body of the product, was calculated by the ratio between the area under the second-bite curve and the area under the first-bite curve. Hardness, defined as the maximum force required to compress the cheese sample 50% from its original height during the first compression. Springiness, defined as the distance regained by the sample during the time between the end of the first compression and the beginning of the second compression. The chewiness was defined as the product of hardness, cohesiveness, and springiness, as described by Bourne [19].

### 2.5. Nitrogenous Fractions Obtention

Nitrogenous fractions were obtained by crude fractionation and were used to evaluate biological activities, antioxidant, and inhibition of the angiotensin-converting enzyme (ACEI) and to evaluate the peptide profile among each fraction. Nitrogen soluble in pH 4.6 (ASN), nonprotein nitrogen (NPN), 70% ethanol-soluble nitrogen (EtOH-SN), and a water-soluble extract (WSE) were obtained. For ASN and NPN the method described by Leclercq-Perlat, et al. [20] was used with minor modifications. A cheese suspension was prepared with 10 g of ground cheese and 100 mL of NaCl solution (9 g/L). This was homogenized (10 min, 25 °C) using an ULTRA-TURRAX IKA T18 basic (Interscience, Wilmington, NC, USA). ASN was obtained by adjusting the pH of the suspension to 4.6 by adding 2N HCl. After pH adjustment, the samples were incubated 20 min at 25 °C. Then, they were centrifuged during 30 min at 6000 rpm. The soluble fraction was recovered after filtration through Whatman No. 42 paper. For NPN, an aliquot of 25 mL of cheese suspension was mixed with 15 mL water. Then 10 mL of 60% (*w*/*v*) trichloroacetic acid (TCA) was added to achieve a final TCA concentration of 12%. Samples were homogenized and incubated at 25 °C for 20 min. Then they were filtered through Whatman No. 42 paper. EtOH-SN was prepared according to the method described by Guerra Martínez, et al. [21]. WSE was prepared according to Rohm, et al. [22], 20 g of cheese was added to 40 mL of distilled water and homogenized for 2 min using an ULTRA-TURRAX IKA T18 basic. The homogenate was held at 40 °C for 1 h and centrifuged at 3000 *g* for 30 min at 4 °C. The fat was removed, then the supernatant was filtered through Whatman No. 42 paper. Nitrogenous fractions were held at −80 °C until analysis. 

### 2.6. Evaluation of Biological Activities of Nitrogenous Fractions

#### 2.6.1. Antioxidant Capacity by ABTS

Antioxidant capacity was measured using the methodology of Re, et al. [23], through the decolorization of the radical 2,2′-azino-bis(3-ethylbenzothiazoline-6-sulphonic acid) (ABTS) detected spectrophotometrically at 734 nm.

A solution of 7 mM radical cation ABTS in a 2.45 mM potassium persulfate solution was prepared and allowed to stand in darkness at room temperature for 16 h. ABTS solution was diluted with ethanol in a 1:20 ratio to get an absorbance of 0.70 (± 0.02) at 734 nm. 20 µL of sample/Trolox and 200 µL of ABTS solution were added to each well, and after 6 min of reaction, absorbance was recorded at 734 nm. Results are expressed as µM equivalents of Trolox.

#### 2.6.2. Antioxidant Activity by DPPH

Anti-free radical activity using 2,2-diphenyl-1-picrylhydrazyl (DPPH) was determined by the method described by Pyrzynska and Pękal [24]. A solution of 125 µM DPPH with 80% methanol was prepared. 20 µL of sample/standard and 200 µL of DPPH were plated in each well and incubated for 90 min in darkness. Absorbance was measured at 520 nm. Results are expressed in % discoloration.

#### 2.6.3. Angiotensin-Converting Enzyme Inhibitory Activity

Evaluation of inhibition of the angiotensin-converting enzyme (ACE) was developed according to the methodology proposed by Wang et al. [25]. ACE (0.1 U/mL mM) and hippuryl histidyl leucine (HHL, 5 mM) were dissolved in borate buffer (100 mM, pH 8.3, 300 mM NaCl).

The reaction mixture (10 µL HHL, 10 µL ACE, 40 µL sample, and 40 µL borate buffer) was incubated at 37 °C for 30 min, and then 250 µL HCl 1N was added to stop the reaction. Samples were analyzed in an HPLC (1200 Agilent, Milford, MA, USA) equipped with an Eclypse XDB-C18 column (4.6 × 150 mm, 5 µm, Agilent). The mobile phase consisted of solvent A, 0.05% TFA (trifluoroacetic acid) and 0.05% TEA (triethylamine) in water; solvent B, 100% ACN (acetonitrile); the ratio of solvent A/solvent B was 7/3 with a gradient of 5–60% of B the first 10 min, 2 min at 60% of B and 1 min of 5% of B. The flow rate was 0.5 mL/min, and the injection volume was 10 µL. The detector was set at 226 nm. The column temperature was held at 30 °C. The inhibitory rate was calculated by:

(1) $$\%I=\frac{\left(A-B\right)}{A}\times 100$$

where *A* was the peak area of HA without adding ACE inhibitors, *B* was the peak area of HA with adding ACE inhibitors.

### 2.7. Peptide Profile by HPLC

The peptide profile was analyzed with the methodology of Abadía-García, et al. [26]. All nitrogenous fractions were analyzed by RP-HPLC. Peptides separation was performed at 25 °C in an Agilent 1200 series system (Agilent Technologies, Palo Alto, Santa Clara, CA, USA) using a Zorbax 300 SB column (C18 5 µm, 4.6 × 150 mm). Mobile phase was solvent A, 10% ACN with 0.05% TFA; solvent B, 60% ACN with 0.05% TFA. The flow rate was 0.75 mL/min. The gradient consisted of 100% of A for 10 min; 0–49% of B from minute 11–98; 50.80% of B from 99–108 min; 81–100% of B from 109–114 min and from 115–120 min 100% of B. The detector was set at 215 nm.

Peaks in each fraction were coded by retention time, and consecutive numbers were assigned for further statistical analysis. Quantification was done using the peak integrated area.

### 2.8. Statistical Analysis

The statistical analyses were carried out using Statistica v13 (TIBCO Software Inc., Palo Alto, CA, USA). One-way analysis of variance (ANOVA) was used to determine significant differences (*p* < 0.05) between the sampling days for antioxidant activity, ACEI, moisture, pH, and TPA parameters of cheeses. General linear model was used to obtain the least square average. For each significant variable, differences between means were detected using Tukey’s honest significant difference (HSD) test with *α* = 0.05. Correlation analyses between physicochemical and textural parameters and among biological activities and the peaks obtained from the peptide profile were done.

Finally, a principal component analysis (PCA) was applied using all the response variables. PCA is a multivariate statistical method that replaces the original variables with new ones called principal components, making it possible to obtain an overview of the data set information.

## 3. Results and Discussion

### 3.1. Physicochemical and Textural Parameters of Cheeses

Mean values of pH, moisture content, and textural properties are given in Table 1. Overall, pH in LSB-c decreased significantly (*p* < 0.05) from day 0 until day 10, and remained the same until day 15; in C-c, pH decreased significantly (*p* < 0.05) constant until day 15 (pH 4.89). C-c final pH was significantly (*p* < 0.05) lower than LSB-c pH. Hayaloglu, et al. [27], confirmed that *Lactococcus* spp., *S. thermophilus*, and *B. animalis* ssp. *lactis* have extensive activity in cheese acidification. For a cheese without added probiotics, results are similar to those previously reported by Guerra Martínez, et al. [21].

Moisture decreased significantly, along with storage in both LSB-c and C-c. The acidification could have enhanced the expulsion of whey and, in the specific case of LSB-c, internal metabolism of added microorganisms [28].

In general, hardness in LSB-c presented significant differences (*p* < 0.05) from day 0 to day 5 but remained constant until the end of storage. Hardness in C-c was greater (*p* < 0.05) than LSB-c. Panela cheese presents a porous structure, and as moisture decreases, the size of porous spaces increases, causing a decrease in instrumental hardness. Additionally, the decrease in hardness in LSB-c could be explained with the casein matrix hydrolysis caused by the added culture [29]. Souza and Saad [30] reported that Minas fresh cheese supplemented with mesophilic culture presented a significant increase in hardness throughout storage and lower hardness values compared with a control cheese. Buriti, et al. [31], found that Minas fresh cheese added with probiotics presented an increase in hardness. Additionally, Dinakar and Mistry [32], reported that probiotics added to Cheddar cheese showed significant changes in texture without affecting either flavor or appearance in the sensorial analysis. Hardness in C-c remained the same until day 10, and decreased at day 15, which could be related to cheese proteolysis that could be determined by the organoleptic characteristics of the cheese.

### 3.2. Correlations Between Physicochemical and Textural Parameters of Cheeses

The correlations observed between texture and physicochemical parameters for LSB-c and C-c are shown in Table 2 and Table 3, respectively. Hardness showed a high positive correlation with moisture, springiness, and chewiness in LSB-c, it can be attributed to early casein matrix hydrolysis by residual enzymes present in the rennet and also to the proteolytic system of added culture [33]. Springiness and hardness are affected by proteolytic enzymes that act mainly over α_s1_ casein [29,34]. A decrease in hardness also contributes to a decrease in chewiness since it is defined as the effort used to chew food to reduce it to the consistency necessary to swallow it.

Moisture is (*p* < 0.05) negatively correlated with cohesivity (Table 2) and is explained by the fact that as the cheese ripens, it becomes a more cohesive material [35]. Springiness and moisture showed a positive correlation. This result matches with those reported by Osorio Tobón, et al. [35], in Edam cheese. Cohesivity is significantly (*p* < 0.05) and negatively correlated with springiness (Table 2). Thus, if the cheese is more cohesive, proteins within it are hydrolyzed, so it becomes more difficult for the cheese to restore its initial shape after compression.

In Table 3, a positive correlation (*p* < 0.05) between pH and moisture content in C-c was observed. This could be attributed to the influence of water on the ionic environment of the cheese, which induces ionization of the calcium phosphate complexes and the functional groups of the amino acids [36].

Springiness is significantly correlated (*p* < 0.05) with moisture and pH. The crumbling characteristics at high pH of the C-c give it a higher capacity to restore its initial shape after compression when more water is available within the cheese matrix. This agrees with the results reported by Osorio Tobón, et al. [35].

Cohesivity is significantly correlated (*p* < 0.05) with chewiness; an increase in curd particle fusion leads to a firmer and closer structure of the cheese; thus, more bites are needed to disrupt the whole structure of cheese before swallowing [21].

### 3.3. Biological Activities of Nitrogenous Fractions

The results of biological activities presented by nitrogenous fractions are given in Table 4. All the biological activities within all nitrogenous fractions presented significant differences (*p* < 0.001). In NPN fraction, LSB-c and C-c ABTS values were significantly different (*p* < 0.05).

#### 3.3.1. Antioxidant Activity of Nitrogenous Fractions

Both types of cheeses presented significant differences in antioxidant activity. ABTS values oscillated significantly during storage time. This could be attributed to the rate of formation of peptides during proteolysis. Gupta, et al. [37], observed the same behavior of a cheddar cheese added with Lactobacilli. NPN showed the highest (*p* < 0.05) percentage of DPPH discoloration during storage. In LSB-c, it increased significantly (*p* < 0.05) from day 0 to day 5, and it remained the same until the end of storage (50.81%); in C-c, it increased significantly (*p* < 0.05) until a 49.49% at day 15. These results are similar to Hernández Galán, et al. [38], who reported the highest discolorations in the NPN fraction of a Cotija hard cheese.

ASN presented the best (*p* < 0.05) ABTS antioxidant activity compared to all the fractions in both LSB-c and C-c; ABTS values remained the same (*p* < 0.05) from day 0 to day 10, and it increased significantly on day 15 for both kinds of cheese (Table 4). C-c ABTS values were lower than those for LSB-c (1511.12 and 1272.08 µM Trolox equivalents, respectively). ASN did not show DPPH free radical scavenging activity. Floegel et al. [39], reported ABTS as the best method for detecting antioxidant capacity in a variety of foods.

ETOH-SN ABTS values in both kinds of cheese were 250 µM Trolox, approximately. These results were similar to those obtained by Abadía-García, et al. [26], who reported an overall antioxidant activity of 300 µM Trolox equivalents in Cottage cheese.

In WSE, ABTS activity for both LSB-c and C-c showed a similar tendency than the other nitrogenous fractions.

#### 3.3.2. Angiotensin Converting Enzyme Activity (ACEI) of Nitrogenous Fractions

NPN fraction also showed significant ACEI activity (*p* < 0.001). In LSB-c, ACEI remained the same (*p* < 0.05) during storage until it increased considerably (*p* < 0.05) at day 15, reaching 90.21%; C-c showed the same tendency (*p* < 0.05) but with a lower final value (80.99%). The ACEI activity could also be related to the release of bioactive peptides during cheese proteolysis. Hernández Galán, et al. [38], evaluated Cotija hard cheese during ripening, and they correlated ACE inhibitory activity with cheese proteolysis.

In ASN, LSB-c presented an average ACEI activity of 45.90% throughout storage. In C-c, ACEI activity was observed after day 5 and remained at 40.06% until the end of storage.

WSE of LSB-c had the highest ACE inhibitory activity (*p* < 0.05) among all the nitrogenous fractions. It is supposed that WSE contains all the peptides produced during proteolysis. This is confirmed by Gorostiza, et al. [40], who reported that the water-soluble extract refers to all the nitrogenous fractions.

### 3.4. Peptide Profile

**Figure 1:**
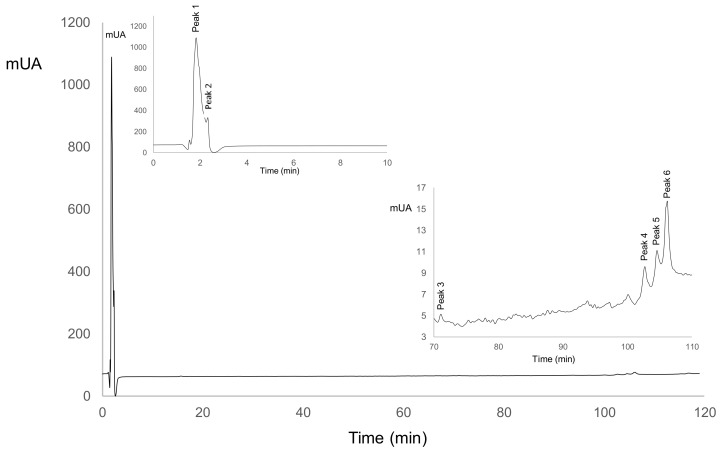
Peptide profile in ASN (nitrogen-soluble at pH 4.6) fraction for LSB-c (probiotic cheese).

Figure 1 shows a representative HPLC chromatogram of the whole experiment, which corresponds to ASN fraction in LSB-c. Table 5 shows peaks and their corresponding peak area count (which is a measure of the concentration of the compound it represents) during storage for both LSB-c and C-c. For each nitrogenous fraction, the significant statistical correlations with biological activities are shown in the corresponding tables (Table 6, Table 7, Table 8 and Table 9).

For ASN, Table 5 shows that in LSB-c, peak 1 and 5 increased their peak area and disappeared on day 15. For C-c, peak 1 appeared at day 5 and increased its peak area until the end of storage. Peak 5 was not present in C-c. Both peaks are positively correlated with ABTS, and Peak 1 is also positively correlated with ACEI (Table 6). Peak 5 could be related to LSB-c ABTS activity. Peak 1 could also be related to ACEI activity since its increase in area is related to an increase in ACEI. Peak 2 decreased over time, but it showed a positive correlation with ABTS and ACE. Peak 3, 4, and 6 were only present on day 0, and in Table 6, it could be observed their positive correlation with ABTS and ACEI. It is suggested that probiotics hydrolyzed those peptides into shorter ones during storage, and thus biological activities increased.

Correlations for the NPN fraction (Table 7) showed a negative correlation of peak 1 with DPPH and ACEI. This peak decreased during storage days (Table 5), while biological activities increased. It could be suggested that probiotics continued enhancing proteolysis, thus even though peak 1 decreased, other peptides were released, and biological activities increased. The positive correlation of day and peak 2 with DPPH and ACEI is confirmed in Table 5, where an increase can be observed during storage.

Table 8 shows the peak’s correlation of ETOH-SN fraction; peak 1, 3, 4, and 5 increased over time (Table 5) and are positively correlated with DPPH; peak 4 is also positively correlated with ABTS. This suggests that their increase during storage is attributed to the increase in antioxidant activity. Peak 2 was not in LSB-c (Table 5), but its positive correlation with ABTS and DPPH suggests that when it appeared in C-c (day 10) was in big quantity (area under the curve), and its increase contributed to the same behavior of antioxidant activities.

In Table 9, it can be observed the correlations of the peaks present within WSE fraction. Peak 1 showed a negative correlation with ACEI; this peak oscillated over time and increased at the end of storage. This suggests that it became partially hydrolyzed, and it increased was not enough to enhance ACEI activity. Peak 2 increased over time (Table 5) and showed a significant positive correlation with ABTS and negative with DPPH. This could suggest that this peak had hydrophilic characteristics; thus, the DPPH was diminished. Peak 3 disappeared from day 5 and 10 and increased on day 15 with a lower value than the initial (Table 5). In Table 9, a negative correlation with ACEI is shown, which is suggested by the hydrolysis of the peptide in shorter ones with no ACEI activity and at day 15, with an increment of peak 3, it is suggested they all coexisted, peptide 3 and the shorter ones, thus even the ACEI activity increased—it was not the same as the beginning of storage. Peak 4 decreases over time (Table 5) and its positive correlation with ABTS, and a negative one with ACEI suggests that this peptide had a hydrophilic profile, which enabled it to show the antioxidant but not the inhibitory activity of ACE.

### 3.5. Principal Component Analysis (PCA)

**Figure 2:**
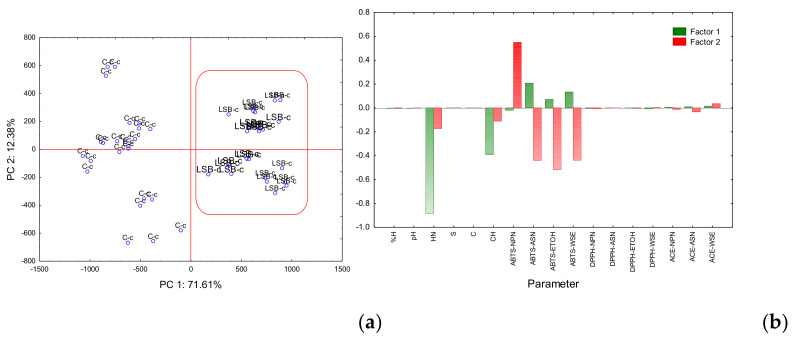
Principal component analysis (PCA) plots for the first two principal components. (**a**) Factorial map: and (**b**) Eigenvectors. %M: % Moisture; HN: Hardness; S: Springiness; C: Cohesivity; CH: Chewiness; ABTS-NPN: ABTS antioxidant activity of Non-Protein Nitrogen; ABTS-ASN: ABTS antioxidant activity of Nitrogen soluble in pH 4.6; ABTS-ETOH: ABTS antioxidant activity of Ethanol-Soluble Nitrogen; ABTS-WSE: ABTS antioxidant activity of Water-Soluble Extract; DPPH-NPN: DPPH radical scavenging of Non-Protein Nitrogen; DPPH-ASN: DPPH radical scavenging of Nitrogen soluble in pH 4.6; DPPH-ETOH: DPPH radical scavenging of Ethanol-Soluble Nitrogen; DPPH-WSE: DPPH radical scavenging of Water-Soluble Extract; ACE-NPN: Angiotensin-Converting Enzyme of Non-Protein Nitrogen; ACE-ASN: Angiotensin-Converting Enzyme of Nitrogen soluble in pH 4.6; ACE-WSE: Angiotensin-Converting Enzyme of Water-Soluble Extract.

The principal component analysis (PCA) plots for the first two principal components is shown in Figure 2a Factorial map and Figure 2b Eigenvectors.

As observed in Figure 2a, the factorial map formed by PC1 and PC2 explained 71.61% and 12.38% of the total variance, respectively. Samples were separated into two well-defined groups by PC1. On the positive side, cheeses with probiotics LSB-c (coded b), and in the negative side, the control cheeses C-c (coded c).

Figure 2b shows that for eigenvector 1, hardness and chewiness showed the highest capacity to separate cheeses in two well-defined groups. Those two variables showed significant differences (*p* < 0.05) within ANOVA (Table 1). ABTS-ASN, ABTS-ETOH, and ABTS-WSE also showed the capacity to separate LSB-c into the positive axis suggesting that probiotic cheese has higher antioxidant activity compared to C-c.

PCA did not make it possible to classify cheeses according to the storage time. Solieri, et al. [41], properly used PCA when studying ripened Parmigiano Reggiano cheese.

## 4. Conclusions

ANOVA made it possible to determine that parameters were affected by the addition of probiotics to Panela cheese. PCA separated samples into two different groups corresponding to LSB-c and C-c, which could be explained by the textural, physicochemical, and biological changes during storage. The addition of probiotics made it possible to increase the biological activities that could have a benefit in the consumer’s health, not only because of the probiotics but also by bioactive peptides released. Correlation between the peaks of the peptide profile and biological activities could be made. A deeper study is necessary to obtain detailed information regarding the identity of peptides, free fatty acids profile, aroma compounds, and sensory attributes. It would also be interesting to evaluate how consumers perceive changes evaluated in this study in a sensory panel evaluation.

## Figures and Tables

**Table 1:** Mean values of physicochemical and textural parameters of LSB-c and C-c both studied during 15 days of storage under refrigeration at 4 ± 0.6 °C.

	Treatments ^b^								
		LSB-c ^¥^				C-c ^¥^			
Parameter ^c^	*p* ^a^	Day 0	Day 5	Day 10	Day 15	Day 0	Day 5	Day 10	Day 15
pH	0.00 ***	4.99 ^a^	4.95 ^b^	4.93 ^c^	4.92 ^c^	6.5 ^d^	6.16 ^e^	6.13 ^e^	4.89 ^f^
Moisture ^s^	0.00 ***	72.11 ^a^	61.10 ^b^	58.95 ^c^	58.12 ^d^	60.95 ^d^	60.53 ^d^	59.77 ^e^	59.17 ^f^
Hardness ^u^	0.00 ***	955.83 ^a^	618.33 ^b^	500.83 ^b^	625.00 ^b^	1720.83 ^e,f^	1883.33 ^d,e^	2068.33 ^d^	1654.17 ^f^
Springiness	0.58	0.92	0.71	0.74	0.69	0.88	0.83	0.84	0.83
Cohesiviness	0.00 ***	0.35 ^b^	0.64 ^a^	0.66 ^a^	0.58 ^a^	0.64 ^d^	0.36 ^e^	0.42 ^e,f^	0.60 ^d,e^
Chewiness ^u^	0.00 ***	308.42 ^a^	275.57 ^a^	243.96 ^a^	247.83 ^a^	971.24 ^d^	565.72 ^e^	741.44 ^d,e^	822.19 ^d,e^

^¥^ LSB-c: Probiotic cheese; C-c: Control cheese. ^a^ Significant at *** *p* < 0.001. ^b^ Means of each cheese with different letters within the same row are significantly different (*p* < 0.05). ^c^ Expressed as: ^s^ %. ^u^ (N).

**Table 2:** Correlation coefficients for the physicochemical and textural parameters analyzed in LSB-c and studied during storage under refrigeration at 4 °C.

Variables ^a^	Moisture ^s^	pH	Hardness	Springiness	Cohesivity
pH	0.23				
Hardness ^u^	0.76 *	0.08			
Springiness	0.97 *	0.29	0.73 *		
Cohesivity	−0.80 *	−0.41 *	−0.79 *	−0.80 *	
Chewiness ^u^	0.36	−0.30	0.65 *	0.33	−0.10

LSB-c: probiotic cheese. * Correlations are significant at *p* < 0.05. ^a^ Expressed as: ^s^ %; ^u^ (N).

**Table 3:** Correlation coefficients for the physicochemical and textural parameters analyzed in C-c and studied during storage under refrigeration at 4 °C.

Variables ^a^	Moisture ^s^	pH	Hardness	Springiness	Cohesivity
pH	0.83 *				
Hardness ^u^	0.06	0.37			
Springiness	0.69 *	0.61 *	−0.25		
Cohesivity	0.06	−0.18	−0.33	0.44 *	
Chewiness ^u^	0.15	0.02	0.00	0.45 *	0.94 *

C-c: control cheese. C-c stands for control cheese. * Correlations are significant at *p* < 0.05. ^a^ Expressed as: ^s^ %; ^u^ (N).

**Table 4:** Mean values of biological activities of nitrogenous fractions analyzed in LSB-c and C-c both studied during storage under refrigeration at 4 °C.

Nitrogenous Fractions ^b^													
NPN					ASN			ETOH-SN			WSE		
*p* ^a^		0.00 ***	0.00 ***	0.00 ***	0.00 ***	0.00 ***	0.00 ***	0.00 ***	0.00 ***	0.00 ***	0.00 ***	0.00 ***	0.00 ***
Tx ^c^	Day	ABTS ^s^	DPPH ^u^	ACE ^w^	ABTS ^s^	DPPH ^u^	ACE ^w^	ABTS ^s^	DPPH ^u^	ACE ^w^	ABTS ^s^	DPPH ^u^	ACE ^w^
LSB-c ^y^	0	708.75 ^c^	32.11 ^b^	60.97 ^b^	1343.33 ^b^	0.00 ^a^	53.11 ^a^	258.33 ^b^	2.51 ^a^	0.00 ^a^	1136.25 ^a,b^	0.00 ^a^	87.35 ^a^
	5	942.50 ^b^	49.11 ^a^	61.16 ^b^	1473.33 ^a,b^	0.00 ^a^	39.04 ^a^	231.25 ^b^	0.00 ^b^	0.00 ^a^	1107.08 ^a,b^	0.00 ^a^	60.17 ^b^
	10	604.58 ^d^	48.09 ^a^	53.87 ^b^	1479.17 ^a,b^	0.00 ^a^	39.42 ^a^	580.83 ^a^	0.00 ^b^	0.00 ^a^	1062.08 ^b^	0.00 ^a^	94.32 ^a^
	15	1152.5 ^a^	50.81 ^a^	90.21 ^a^	1511.12 ^a^	0.00 ^a^	52.07 ^a^	230.58 ^b^	2.57 ^a^	0.00 ^a^	1336.25 ^a^	0.00 ^a^	76.39 ^a,b^
C-c ^z^	0	959.58 ^a^	37.33 ^c^	49.79 ^b^	924.17 ^b^	0.00 ^b^	0.00 ^b^	239.58 ^b^	0.00 ^b^	0.00 ^a^	584.17 ^b^	6.18 ^b^	56.86 ^b^
	5	890.83 ^b^	44.56 ^b,c^	63.96 ^b^	1082.92 ^a,b^	0.00 ^b^	46.37 ^a^	242.08 ^b^	0.00 ^b^	0.00 ^a^	994.58 ^a^	0.00 ^b^	66.06 ^a,b^
	10	927.92 ^a,b^	48.25 ^a,b^	45.49 ^b^	1073.33 ^a,b^	1.10 ^b^	29.82 ^a,b^	256.67 ^b^	0.00 ^b^	0.00 ^a^	1060.00 ^a^	36.81 ^a^	75.22 ^a^
	15	596.67 ^c^	49.49 ^a^	80.99 ^a^	1272.08 ^a^	4.29 ^a^	57.79 ^a^	354.17 ^a^	3.76 ^a^	0.00 ^a^	1200.00 ^a^	0.00 ^b^	0.00 ^c^

LSB-c: probiotic cheese; C-c: control cheese. ^a^ Significant at *** *p* < 0.001. ^y/z^ Means with different letters within the same column are significantly different (*p* < 0.05). ^b^ NPN: Non-protein nitrogen, ASN: Nitrogen soluble at pH 4.6, ETOH-SN: 70% ethanol-soluble nitrogen, WSE: Water-soluble extract. ^c^ Tx: Treatment. Expressed as: ^s^ ABTS (2,2’-Azino-bis(3-ethylbenzothiazoline-6-sulfonic acid) diammonium salt) uM Trolox equivalents; ^u^ DPPH (2,2-diphenil-1-picrylhydrazyl) % discoloration; ^w^ ACE (Angiotensin converting enzyme) % inhibition.

**Table 5:** Peak Area within each nitrogenous fraction was analyzed in LSB-c and C-c during storage under refrigeration at 4 °C.

			Peak Area ^b^							
			Day 0		Day 5		Day 10		Day 15	
NF ^c^	Peak number	Retention time ^a^	LSB-c ^¥^	C-c ^¥^	LSB-c	C-c	LSB-c	C-c	LSB-c	C-c
ASN	1	2.005	5562	0	8707	5030	10,022	6123	0	7231
	2	2.245	3772	0	2432	0	2300	763	2158	659
	3	72.428	58	0	0	0	0	0	0	0
	4	102.088	374	0	0	0	0	0	0	0
	5	102.935	11	0	339	0	333	0	128	0
	6	105.52	841	0	0	0	0	0	0	0
NPN	1	4.468	5137	5019	5076	5239	5296	5155	4588	4049
	2	116.899	0	221	220	345	384	420	610	568
ETOH-SN	1	1.536	21	20	39	25	34	35	112	74
	2	1.742	0	0	0	0	0	2101	0	6463
	3	1.842	5780	2547	5800	3504	6549	0	6960	0
	4	2.597	14	12	13	14	15	17	17	26
	5	116.653	377	220	180.6	178	203	230	451	345
WSE	1	1.12	533	509	556	570	483	475	596	576
	2	1.889	52,003	18,050	58,595	51,901	70,450	30,852	75,957	60,113
	3	2.286	3800	1759	0	0	0	5223	1688	4305
	4	117.284	690	676	754	680	621	325	345	301

^a^ Expressed in minutes. ^b^ Expressed as units of chromatogram area. ^c^ NF: Nitrogenous fraction: ASN: Nitrogen soluble at pH 4.6; NPN: Non-protein nitrogen; ETOH-SN: Nitrogen soluble in Ethanol; WSE: Water-soluble extract. ^¥^ LSB-c: probiotic cheese; C-c: control cheese.

**Table 6:** Correlation coefficients for the peaks, day, treatment (LSB-c and C-c), and the biological activity parameters analyzed in ASN fraction of Panela cheeses studied during storage under refrigeration at 4 °C.

Peak						
Parameter ^a^	1 (2.005) ^r^	2 (2.245)	3 (72.428)	4 (102.088)	5 (102.935)	6 (103.520)
Treatment	−0.28 *	−0.61 ***	−0.32 *	−0.32 *	−0.5 ***	−0.32 **
Day	−0.28 *	−0.14	−0.43 ***	−0.43 ***	−0.01	−0.43 ***
ABTS ^s^	0.39 **	0.5 ***	0.38 **	0.37 **	0.43 ***	0.39 **
DPPH ^u^	−0.08	−0.24	−0.14	−0.14	−0.23	−0.15
ACE ^w^	0.52 ***	0.3 *	0.34 **	0.31 *	0.11	0.32 **

LSB-c: Probiotic cheese; C-c: Control cheese; ASN: Nitrogen soluble at pH 4.6. ^a^ Correlations are significant at * *p* < 0.05, ** *p* < 0.01, *** *p* < 0.001. Expressed as: ^s^ ABTS (2,2’-Azino-bis(3-ethylbenzothiazoline-6-sulfonic acid) diammonium salt) uM Trolox equivalents; ^u^ DPPH (2,2-diphenil-1-picrylhydrazyl) % discoloration; ^w^ ACE (Angiotensin converting enzyme) % inhibition. ^r^ Numbers in brackets: retention time.

**Table 7:** Correlation coefficients for the peaks area, day, treatment (LSB-c and C-c), and the biological activity parameters analyzed in NPN fraction of Panela cheeses studied during storage under refrigeration at 4 °C.

Peak ^r^		
Parameter ^a^	1 (4.468)	2 (116.899)
Treatment	−0.05	0.19
Day	−0.51 ***	0.92 ***
ABTS ^s^	−0.07	−0.05
DPPH ^u^	−0.35 **	0.75 ***
ACE ^w^	−0.55 ***	0.61 ***

LSB-c: probiotic cheese; C-c: control cheese; NPN: Non protein nitrogen. ^a^ Correlations are significant at ** *p* < 0.01, *** *p* < 0.001. Expressed as: ^s^ uM Trolox equivalents; ^u^ % discoloration; ^w^ ACE: Angiotensin converting enzyme % inhibition. ^r^ Numbers in brackets: retention time.

**Table 8:** Correlation coefficients for the peaks, day, treatment (LSB-c and C-c), and the biological activity parameters analyzed in ETOH-SN fraction of Panela cheeses studied during storage under refrigeration at 4 °C.

Peak ^r^					
Parameter ^a^	1 (1.536)	2 (1.742)	3 (1.842)	4 (2.597)	5 (116.653)
Treatment	−0.23	0.43 ***	−0.66 ***	0.41 **	−0.19
Day	0.6 ***	0.53 ***	−0.05	0.49 ***	0.32 *
ABTS ^s^	0.1	0.54 ***	−0.03	0.37 **	0.24
DPPH ^u^	0.51 ***	0.64 ***	−0.28 *	0.58 ***	0.67 ***

LSB-c: Probiotic cheese; C-c: Control cheese; ETOH-SN: Nitrogen soluble in ethanol. ^a^ Correlations are significant at * *p* < 0.05, ** *p* < 0.01, *** *p* < 0.001. Expressed as: ^s^ ABTS (2,2’-Azino-bis(3-ethylbenzothiazoline-6-sulfonic acid) diammonium salt) uM Trolox equivalents; ^u^ DPPH (2,2-diphenil-1-picrylhydrazyl) % discoloration;. ^r^ Numbers in brackets: Retention time.

**Table 9:** Correlation coefficients for the peaks, day, treatment (LSB-c and C-c) and the biological activity parameters analyzed in WSE fraction of Panela cheeses studied during storage under refrigeration at 4 °C.

Peak ^r^				
Parameter ^a^	1 (1.120)	2 (1.889)	3 (2.286)	4 (117.284)
Treatment	0.42 ***	−0.45 ***	0.38 **	−0.15
Day	−0.02	0.34 **	−0.02	−0.75 ***
ABTS ^s^	−0.13	0.54 ***	−0.22	−0.6 ***
DPPH ^u^	0.03	−0.57 ***	0.25	−0.05
ACE ^w^	−0.39 **	0	−0.36 **	0.54 ***

LSB-c: Probiotic cheese; C-c: Control cheese; WSE: Water soluble extract. ^a^ Correlations are significant at ** *p* < 0.01, *** *p* < 0.001. Expressed as: ^s^ ABTS (2,2’-Azino-bis(3-ethylbenzothiazoline-6-sulfonic acid) diammonium salt) uM Trolox equivalents; ^u^ DPPH (2,2-diphenil-1-picrylhydrazyl) % discoloration; ^w^ ACE (Angiotensin converting enzyme) % inhibition. ^r^ Numbers in brackets: Retention time.

## References

[1] Gobbetti M., Minervini F., Rizzello C.G. (2004). Angiotensin I-converting-enzyme-inhibitory and antimicrobial bioactive peptides. Int. J. Dairy Technol..

[2] Moller N.P., Scholz-Ahrens N.R., Schrezenmeir J. (2008). Bioactive peptides and proteins from foods: Indication for health effects. Eur. J. Nutr..

[3] Korhonen H., Pihlanto A. (2006). Bioactive peptides: Production and functionality. Int. Dairy J..

[4] Korhonen H. (2009). Milk-derived bioactive peptides: From science to applications. J. Funct. Foods.

[5] Rasmusson K. (2012). Bioactive Peptides in long-Time Ripened Open Texture Semi-Hard Cheese. Master’s Thesis.

[6] Park Y.W., Nam M.S. (2015). Bioactive Peptides in Milk and Dairy Products: A Review. Korean J. Food Sci. Anim. Resour..

[7] O’Brien N.M., O’Connor T.P., McSweeney P.L.H., Fox P.F., Cotter P.D., Everett D.W. (2017). Nutritional Aspects of Cheese. Cheese.

[8] SAGARPA (2018). Panorama de la Lechería en México [Overview of the Dairy in Mexico].

[9] Cervantes Escoto F. (2008). Los Quesos Mexicanos Genuinos. Patrimonio Cultural Que Debe Rescatarse.

[10] Farkye N.Y., Vedamuthu E., Products M., Robinson R.K. (2002). Microbiology of soft cheeses. Dairy Microbiology Handbook: The Microbiology of Milk and Milk Products.

[11] Hayes M., Stanton C., Fitzgerald G.F., Ross R.P. (2007). Putting microbes to work: Dairy fermentation, cell factories and bioactive peptides. Part II: Bioactive peptide functions. Biotechnol. J..

[12] Campos-Nonato I., Hernández-Barrera L., Pedroza-Tobías A., Medina C., Barquera S. (2018). Hipertensión arterial en adultos mexicanos: Prevalencia, diagnóstico y tipo de tratamiento. Ensanut MC 2016. Salud Pública de México.

[13] Baró L., Jiménez J., Martínez-Férez A., Bouza J. (2017). Péptidos y proteínas de la leche con propiedades funcionales. Ars. Pharm. (Internet).

[14] W.H.O. Cardiovascular Diseases (CVDs) Fact Sheet. http://www.who.int/mediacentre/factsheets/fs317/en/.

[15] NOM-116-SSA1-1994 (1995). Bienes y servicios. Determinación de Humedad en Alimentos por Tratamiento Térmico. Método por Arena o Gasa.

[16] NMX-F-317-S-1978 (1978). Determinación de pH en alimentos. Determinatizon of pH in Fozods.

[17] Voisey P.W., Crete R. (1973). A technique for establishing instrumental conditions for measuring food firmness to simulate consumer evaluations. J. Texture Stud..

[18] Hernández M., Sariñana A. (2007). Diseño Integral de Máquina General de Ensayos de Materiales. 5º Congreso Internacional Sobre Investigación y Desarrollo Tecnológico (CIINDET).

[19] Bourne M. (2002). Food Texture and Viscosity: Concept and Measurement.

[20] Leclercq-Perlat M.N., Oumer A., Bergère J.L., Spinnler H.E., Corrieu G. (1999). Growth of Debaryomyces hansenii on a bacterial surface-ripened soft cheese. J. Dairy Res..

[21] Guerra Martínez J.A., Montejano J.G., Martin del Campo S.T. (2012). Evaluation of proteolytic and physicochemical changes during storage of fresh Panela cheese from Queretaro, Mexico and its impact in texture. CYTA J. Food.

[22] Rohm H., Jaros D., Rockenbauer C., Riedler-Hellrigl M., Uniacke-Lowe T., Fox P. (1996). Comparison of ethanol and trichloracetic acid fractionation for measurement of proteolysis in Emmental cheese. Int. Dairy J..

[23] Re R., Pellegrini N., Proteggente A., Pannala A., Yang M., Rice-Evans C. (1999). Antioxidant activity applying an improved ABTS radical cation decolorization assay. Free Radic. Bio Med..

[24] Pyrzynska K., Pękal A. (2013). Application of free radical diphenylpicrylhydrazyl (DPPH) to estimate the antioxidant capacity of food samples. Anal. Methods.

[25] Wang W., Wang N., Zhang Y., Cai Z., Chen Q., He G. (2013). A Convenient RP-HPLC Method for Assay Bioactivities of Angiotensin I-Converting Enzyme Inhibitory Peptides. ISRN Biotechnol..

[26] Abadía-García L., Cardador A., Martín del Campo S.T., Arvízu S.M., Castaño-Tostado E., Regalado-González C., García-Almendarez B., Amaya-Llano S.L. (2013). Influence of probiotic strains added to Cottage Cheese on potentially-antioxidant peptides generation, anti-Listerial activity, and survival of probiotic microorganisms in simulated gastrointestinal conditions. Int. Dairy J..

[27] Hayaloglu A.A., Guven M., Fox P.F., Hannon J.A., McSweeney P.L.H. (2004). Proteolysis in Turkish White-brined cheese made with defined strains of *Lactococcus*. Int. Dairy J..

[28] Gomez M.J., Gaya P., Nunez M., Médina M. (1998). *Streptococcus thermophilus* as adjunct culture for a semi-hard cows’ milk cheese. Lait.

[29] Lawrence R.C., Creamer L.K., Gilles J. (1987). Texture Development During Cheese Ripening. J. Dairy Sci..

[30] Souza C.H.B., Saad S.M.I. (2009). Viability of *Lactobacillus acidophilus* La-5 added solely or in co-culture with a yoghurt starter culture and implications on physico-chemical and related properties of Minas fresh cheese during storage. LWT Food Sci. Technol..

[31] Buriti F.C.A., da Rocha J.S., Assis E.G., Saad S.M.I. (2005). Probiotic potential of Minas fresh cheese prepared with the addition of *Lactobacillus paracasei*. LWT Food Sci. Technol..

[32] Dinakar P., Mistry V.V. (1994). Growth and Viability of *Bifidobacterium bifidum* in Cheddar Cheese1. J. Dairy Sci..

[33] Bertola N.C., Califano A.N., Bevilacqua A.E., Zaritzky N.E. (1996). Textural Changes and Proteolysis of Low-Moisture Mozzarella Cheese Frozen under Various Conditions. LWT Food Sci. Technol..

[34] Lucey J.A., Johnson M.E., Horne D.S. (2003). Invited Review: Perspectives on the Basis of the Rheology and Texture Properties of Cheese. J. Dairy Sci..

[35] Osorio Tobón J.F., Ciro Velásquez H.J., Mejía Restrepo L.G. (2004). Caracterización textural y fisicoquímica del queso Edam. Rev. Fac. Nac. Agron. Medellín.

[36] Lee S.K., Anema S., Klostermeyer H. (2004). The influence of moisture content on the rheological properties of processed cheese spreads. Int. J. Food Sci. Technol..

[37] Gupta A., Mann B., Kumar R., Sangwan R.B. (2009). Antioxidant activity of Cheddar cheeses at different stages of ripening. Int. J. Dairy Technol..

[38] Hernández Galán L., Cardador Martínez A., Picque D., Spinnler H.E., López del Castillo Lozano M., Martín del Campo Barba S.T. (2016). Angiotensin converting enzyme inhibitors and antioxidant peptides release during ripening of Mexican Cotija hard cheese. J. Food Res..

[39] Floegel A., Kim D.-O., Chung S.-J., Koo S.I., Chun O.K. (2011). Comparison of ABTS/DPPH assays to measure antioxidant capacity in popular antioxidant-rich US foods. J. Food Compos. Anal..

[40] Gorostiza A., Cichoscki A.J., Valduga A.T., Valduga E., Bernardo A., Fresno J.M. (2004). Changes in soluble nitrogenous compounds, caseins and free amino acids during ripening of artisanal prato cheese; a Brazilian semi-hard cows variety. Food Chem..

[41] Solieri L., Bianchi A., Mottolese G., Lemmetti F., Giudici P. (2014). Tailoring the probiotic potential of non-starter *Lactobacillus* strains from ripened Parmigiano Reggiano cheese by in vitro screening and principal component analysis. Food Microbiol..

